# Activation of PXR by alantolactone ameliorates DSS-induced experimental colitis via suppressing NF-κB signaling pathway

**DOI:** 10.1038/s41598-019-53305-z

**Published:** 2019-11-12

**Authors:** Yijing Ren, Bei Yue, Gaiyan Ren, Zhilun Yu, Xiaoping Luo, Aning Sun, Jingjing Zhang, Mengqing Han, Zhengtao Wang, Wei Dou

**Affiliations:** 1 Shanghai Key Laboratory of Formulated Chinese Medicines, Institute of Chinese Materia Medica, Shanghai University of Traditional Chinese Medicine (SHUTCM), Shanghai, 201203 China; 2 Yan’an University Affiliated Hospital, Shanxi Province, 716000 China

**Keywords:** Receptor pharmacology, Ulcerative colitis

## Abstract

Alantolactone (ALA) is a sesquiterpene lactone with potent anti-inflammatory activity. However, the effect of ALA on intestinal inflammation remains largely unknown. The present study demonstrated that ALA significantly ameliorated the clinical symptoms of dextran sulfate sodium (DSS)-induced mice colitis as determined by body weight loss, diarrhea, colon shortening, inflammatory infiltration and histological injury. In mice exposed to DSS, ALA treatment significantly lowered pro-inflammatory mediators, including nuclear factor-kappa B (NF-κB) activation. *In vitro*, ALA inhibited NF-κB nuclear translocation and dose-dependently activated human/mouse pregnane X receptor (PXR), a key regulator gene in inflammatory bowel disease (IBD) pathogenesis. However, the pocket occluding mutants of the ligand-binding domain (LBD) of hPXR, abrogated ALA-mediated activation of the receptor. Overexpression of hPXR inhibited NF-κB-reporter activity and in this setting, ALA further enhanced the hPXR-mediated inhibition of NF-κB-reporter activity. Furthermore, silencing hPXR gene demonstrated the necessity for hPXR in downregulation of NF-κB activation by ALA. Finally, molecular docking studies confirmed the binding affinity between hPXR-LBD and ALA. Collectively, the current study indicates a beneficial effect of ALA on experimental IBD possibly via PXR-mediated suppression of the NF-κB inflammatory signaling.

## Introduction

Inflammatory bowel disease (IBD), consisting of Crohn’s disease (CD) and ulcerative colitis (UC), is a common chronic nonspecific inflammatory disease of the digestive tract and is usually accompanied by diarrhea, abdominal pain, weight loss and malnutrition, which seriously affects the quality of life^1^. Conventional treatment options for IBD are various, including anti-inflammatory, immunosuppressive and biologic therapy, all of which can improve the symptoms, but have significant side-effects^2,3^. Clearly, there is an unmet and urgent need for developing novel treatment approaches for IBD patients.

As an important ligand-activated transcription factor, pregnane X receptor (PXR, NR1I2) belongs to the superfamily of nuclear receptors, which is responsible for the expression of drug-metabolizing enzymes and transporters^4^. It has been proved that PXR is mainly expressed in the liver and intestines of mammals. Furthermore, PXR possesses a bulky and adaptable ligand-binding pocket, which enables it to bind various types of ligands, such as western medicines, Chinese medicines, food additives, contaminants, endogenous hormones, and bile acids^4,5^. Notably, the ligands of PXR display distinct specificity of the host specie. For example, rifaximin, as a human (h)-specific PXR agonist, affects the activity of cytochrome (CYP) 3A4 enzyme, whereas pregnenolone 16α-carbonitrile (PCN) can only activate rodent PXR.

Accumulating evidence suggests that the biological functions of PXR are not confined to mediate metabolism and degradation of xenobiotic compounds. PXR also plays a pivotal role in the process of certain human disorders including diabetes, cholestasis, hyperlipidemia, cancer and IBD^6^. Recently, some studies showed that activation of PXR protects against chemical-induced IBD in mice, and mutation of PXR gene is highly correlated with the susceptibility to IBD^5,7,8^. Furthermore, studies on the pathogenesis of IBD have revealed that some specific single-nucleotide polymorphisms in PXR gene have been associated with a reduction of PXR expression and an increase of human IBD susceptibility^8^. Additionally, a reciprocal crosstalk has been described between nuclear factor-kappa B (NF-κB) and PXR^9^. Activation of PXR suppresses the expression of NF-κB downstream pro-inflammatory genes; however, activation of NF-κB also down-regulates PXR downstream genes^5,9^. Moreover, as a mouse (m)-specific PXR ligand, PCN can relieve inflammatory injury through inhibiting NF-κB signaling in colitis mice, which suggests the potency of PXR activators as therapeutic agents for IBD patients^10^. In previous study, we have shown that flavonoid baicalein alleviates DSS colitis through the caudal type homeobox 2 (Cdx2)-mediated PXR activation mechanism^11^. An emerging body of research has indicated that treatment with medicinal plants or their active components can offer much more therapeutic options for IBD^2,12^.

Alantolactone (ALA), a natural sesquiterpene lactone and marker compound isolated from *Inula helenium L*. and *Inula japonica*., possesses a various of biological activities such as anti-microbial, anti-allergic, anti-oxidative, anti-oncogenic and neuroprotective activities^13,14^. Several studies have indicated that ALA has a concentration-response relationship in controlling neuroinflammation, which mainly through restraining the activation of NF-κB and mitogen-activated protein kinases (MAPKs)^15,16^. Recently, ALA was reported to improve inflammation-induced insulin resistance and glucose intolerance in skeletal cells through blockade the activation of signal transducer and activator of transcription 3 (STAT3) and the abnormal expression of toll-like receptor-4 (TLR4) induced by pro-inflammatory factor interleukin (IL)-6^17^. Furthermore, a negative regulation mechanism of PXR on TLR4 signaling pathway in intestinal epithelial barrier has been revealed recently^18^. In view of the established evidence, we hypothesized that ALA might ameliorate chemically induced IBD via a mechanism associated with PXR regulation.

## Materials and Methods

### Mice

Healthy female C57BL/6 mice (20 ± 2 g) were purchased from the Shanghai Laboratory Animal Center. All mice were maintained in specific pathogen-free facility and kept under controlled conditions at a humidity of 60–70% and stationary temperature of 23–25 °C with a 12 hr light/dark cycle and with access to autoclaved food and drinking water. All experiments were approved by the Animal Experimentation Ethics Committee at Shanghai University of Traditional Chinese Medicine (SHUTCM), and all of the animal experiments was carried out according to the approved guidelines.

### DSS-induced colitis and assessment of colitis

**Figure 1:**
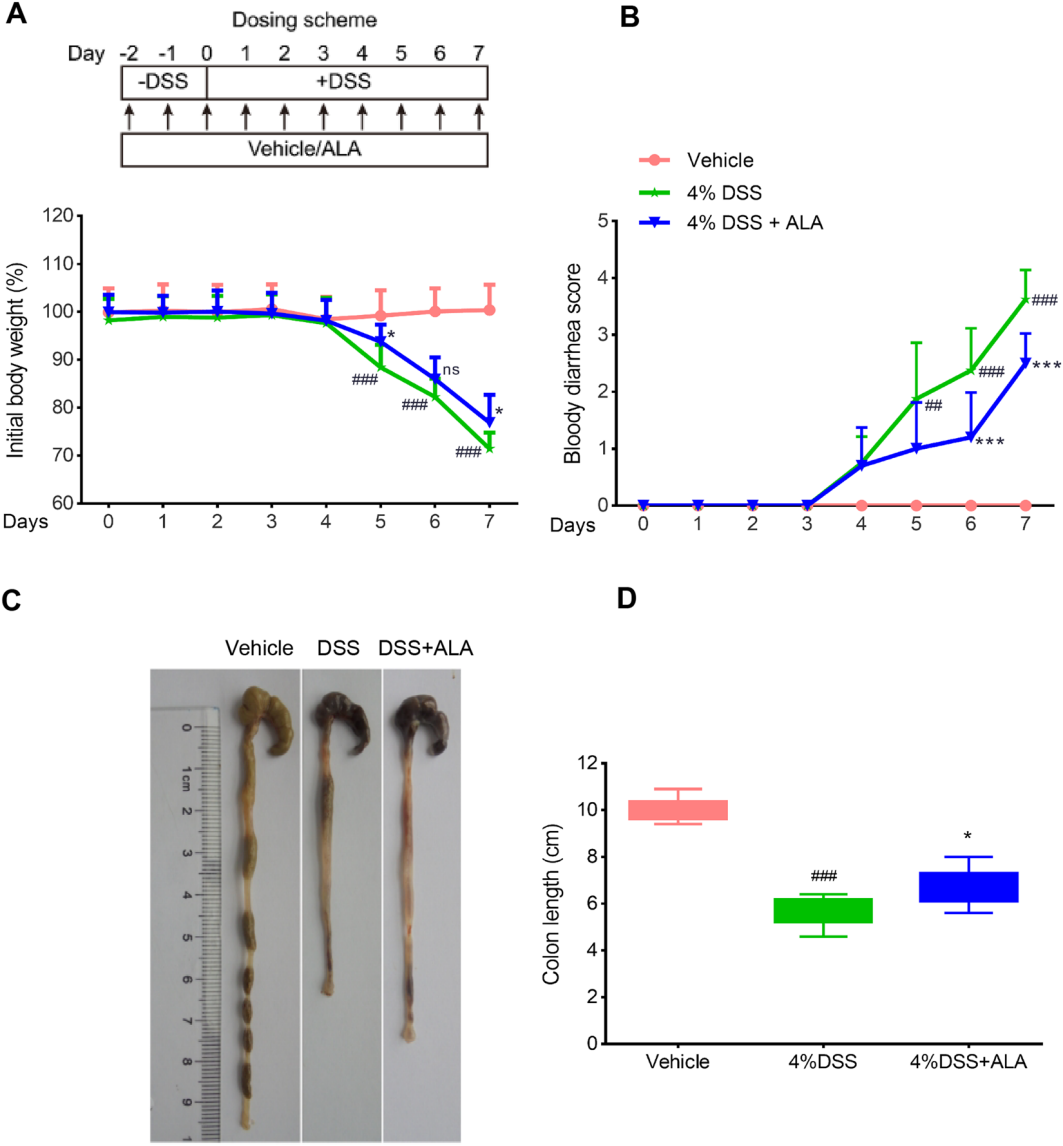
ALA ameliorated body weight loss, bloody diarrhea, and colon shortening in DSS colitis mice. (**A**) Body weight changes following DSS induction of colitis. Data were plotted as a percentage of basal body weight. (**B**) The occurrence of bloody diarrhea. Data were plotted as percentage of total mice that had bloody diarrhea at different time points of DSS treatment. Macroscopic observation (**C**) and assessment of colon shortening (D) at the end of mice study. Data were expressed as the mean ± SD (n = 10 mice per group). *p < 0.05, ***p < 0.001 vs. DSS model group; ^##^p < 0.01, ^###^p < 0.001 vs.control group; ns, no significance.

As described previously^11^, experimental colitis was induced in mice by giving drinking water *ad libitum* containing 4% (w/v) DSS (36–50 kDa, MP Biomedicals, Solon, OH) for 7 days, while control mice received tap water only. ALA (purity ≥98%, HPLC) was dissolved in 0.5% methylcellulose at 50 mg/kg dosage and administered by oral gavage 2 days prior to DSS treatment and continued to the end of the DSS treatment (Fig. 1A, upper panel). ALA was kindly provided by Shanghai R&D Center for Standardization of TCM (Shanghai, China). ALA dosing is referred to previous report and our preliminary studies^19^.

Body weight, diarrhea and bloody stool changes were evaluated and recorded daily. Mice were sacrificed under anesthesia at d 9 after the last gavage. Blood was collected and the spleen weight was recorded. The colon was removed and the colon length was measured. The distal colon was cut off and immediately fixed with 10% formaldehyde and embedded in paraffin. Tissue sections were stained with hematoxylin and eosin (H&E) for histological evaluation. Histological injury was assessed by a combined score of inflammatory cell infiltration (score 0–3) and mucosal damage (score 0–3) as previously described^2,11^.

### *In silico* docking analysis

*The in silico* modeling study was performed as described previously^11^. The ligand affinity was evaluated by setting up the hPXR ligand hyperforin as a template compound. The three-dimensional structure of hPXR co-crystalized with hyperforin was obtained from Protein Data Bank (PDB code: 1M13). The co-crystalized structure was constructed using the MOE (Molecular Operating Environment, version 2012.10, Chemical Computing Group, Montreal, Canada) program. The binding site was characterized based on structural information derived from various co-crystals (PDB Code: 1ILH/1M13/1SKX/2QNV/3R8D). Protein Ligand Interaction Fingerprints (PLIF) program was used to analyze the co-crystals and identify the conserved pocket residues. The constructed structure was submitted to FlexX (BioSolveIT, Germany) procedure for docking analysis. The residues around template ligand hyperforin within 7 Å were selected as the critical binding residues. Subsequently, hyperforin was removed and ALA was docked into the crystal structure. The docking mode was analyzed by MOE program following energy minimization.

### The hPXR knockdown and overexpression assays

HT-29 and LS174T human colorectal carcinoma cells were obtained from American Type Culture Collection (ATCC). For the hPXR knockdown assay, 1 × 10^6^ LS174T cells were electroporated with the hPXR siRNA (sc-44057, Santa Cruz Biotech., CA) or the control siRNA using Lonza Nucleofector II instrument (Amaxa Biosystems, MD). For the hPXR overexpression assay, 1 × 10^6^ HT-29 cells were electroporated with the hPXR expression vector pSG5-hPXR or the pSG5 vacuum vector. The cells were then subjected to immunoblotting analysis or NF-κB luciferase reporter assay.

### Wild-type/mutant PXR transactivation reporter assay

For the wild-type PXR transactivation assay, 1 μg CYP3A4-luciferase reporter combined with 0.1 μg pRL-TK, and 0.5 μg of plasmid expressing wild-type human PXR (pSG5-hPXR) or wild-type mouse PXR (pSG5-mPXR) were co-transfected into 1 × 10^6^ HT-29 cells using Lonza Nucleofector II instrument. For the mutant PXR transactivation assay, 1 μg CYP3A4-luciferase reporter combined with 0.1 μg pRL-TK, and 0.5 μg plasmid expressing the double-mutant (S247W/C284W) or the triple-mutant (S247W/C284W/S208W) hPXR were co-transfected into cells. Previous studies have shown the detailed plasmids information^7,11^. The cells were incubated with ALA (0–25 μM) and rifampicin (10 μM) or ALA (0–25 μM) and PCN (10 μM) for 24 h and were lysed with passive lysis buffer (Promega, Madison, WI). The luciferase activity of cell lysate was analyzed using the dual-luciferase reporter assay system (Promega, Madison, WI). The results are expressed as the fold induction of the control cells.

### NF-κB luciferase reporter assay

The pGL4.32[luc2P/NF-κB-RE/Hygro] luciferase reporter vector (Promega, Madison, WI) was electroporated into LS174T cells or HT-29 cells. The pGL4.32 reporter is a NF-κB reporter vector including NF-κB response elements and firefly luciferase gene. Cells were incubated with ALA (0–25 µM) for 2 h followed by an additional co-incubation with lipopolysaccharide (LPS, 1 µg/ml) for 24 h. The luciferase activity was detected using a luciferase assay system (Promega, Madison, WI).

### Immunoblotting analysis

Colon segments were homogenized in phosphate buffer saline (PBS) and the supernatant was collected after centrifugation (4 °C, 12,000 *g*, 15 min). The cultured cells were lysed in lysis buffer containing protease and phosphatase inhibitor cocktail tablets (Roche Diagnostics, Mannheim, Germany) and the supernatant was collected. The protein (30 µg) in supernatant was separated by 10% SDS-PAGE and transferred onto a PVDF membrane. The membrane was blocked in 5% (w/v) skim milk for 2 h at room temperature and immunoblotted with primary antibodies (Cell Signaling Technology, Danvers, MA) against the mouse p-p65, p-IκBα and IκBα, as well as antibody against the human PXR (Abcam Company, Cambridge, MA), respectively. Blots were incubated with HRP-coupled secondary antibodies (Santa Cruz Biotechnology, Dallas, TX) and observed by enhanced chemiluminescence (ECL) detection reagents (Thermo Scientific, Waltham, MA). Protein expressions were analyzed by a GS-700 imaging densitometer (Bio-Rad, CA). β -actin (Santa Cruz) was used as an internal control.

### RNA analysis

Total RNA extraction from colon samples or cultured cells was performed using TRIzol reagent, and cDNA was reversely transcribed from 3 μg of total RNA using the SuperScript II Reverse Transcriptase kit (Life Technologies, Carlsbad, CA). Quantitative polymerase chain reaction (qPCR) was carried out using SYBR Premix ExTaq Mix (Takara Bio Inc., Otsu, Japan) and quantitatively measured with an ABI Prism 7900HT Sequence Detection System (Life Technologies,s, Carlsbad, CA). The relative expression of mRNA was normalized as the ratio of optimal density relative to β-actin. Primer sequences are as follows (forward 5′-3′, reverse 5′-3′): iNOS (GGGAATCTTGGAGCGAGTTG, GTGAGGGCTTGGCTGAGTGA), COX-2 (GAAGTCTTTGGTCTGGTGCCT, GCTCCTGCTTGAGTATGTCG), intercellular adhesion molecule-1 (ICAM-1) (CGCTGTGCTTTGAGAACTGT, AGGTCCTTGCCTACTTGCTG), monocyte chemotactic protein-1 (MCP-1) (5′-AAGTTGACCCGTAAATCTGA-3′/5′-TGAAAGGGAATACCATAACA-3′), interferon-gamma (IFN-γ) (AGCAACAACATAAGCGTCAT, CCTCAAACTTGGCAATACTC), TNF-α (CGTGGAACTGGCAGAAGAGG, AGACAGAAGAGCGTGGTGGC), IL-6 (ATCCAGTTGCCTTCTTGGGACTGA, TAAGCCTCCGACTTGTGAAGTGGT), and β-actin (CAGCCTTCCTTCTTGGGTAT, TGGCATAGAGGTCTTTACGG).

### Determination the levels of TNF-α IL-6, NO and PGE2 levels

Colon segments were homogenized in PBS and the supernatant was collected after centrifugation (4 °C, 12,000 *g*, 15 min). The levels of TNF-α and IL-6 in the supernatants were measured by mouse-specific enzyme-linked immunosorbent assay (ELISA) kits according to manufacturer’s instructions (R&D systems, Minneapolis, MN). The level of NO in colon tissue was determined using a NO assay kit (Nanjing Jiancheng Bioengineering Institute, China) as described previously^20^. The accumulated PGE2 in serum was measured using a mouse PGE2 ELISA kit (Nanjing Jiancheng Bioengineering Institute, China) according to the manufacturer’s instructions.

### Myeloperoxidase (MPO) assay

MPO activity in colon tissue was measured by MPO detection kit according to the manufacturer’s instructions (Nanjing Jiancheng Bioengineering Institute, China).

### Immunohistochemistry

The phospho-NF-kB p65-NLS immunostaining was performed using paraffin-embedded tissue slides as described previously^21^.

### Immunofluorescence staining

RAW264.7 cells were obtained from ATCC and were exposed to ALA (25 μM) in advance for 2 h and then incubated with LPS (1 μg/mL) for additional 24 h. The cell immunostaining was performed as described previously^21^.

### Statistics

Results were expressed as mean ± SD. The differences between groups were evaluated by one-way ANOVA followed by the least significant difference (LSD) for post-hoc test. For Statistical analysis, the SPSS 16.0 software package was performed and p-values < 0.05 (two-sided) were considered statistically significant.

## Results

### ALA ameliorated clinical symptoms of DSS-induced colitis

**Figure 2:**
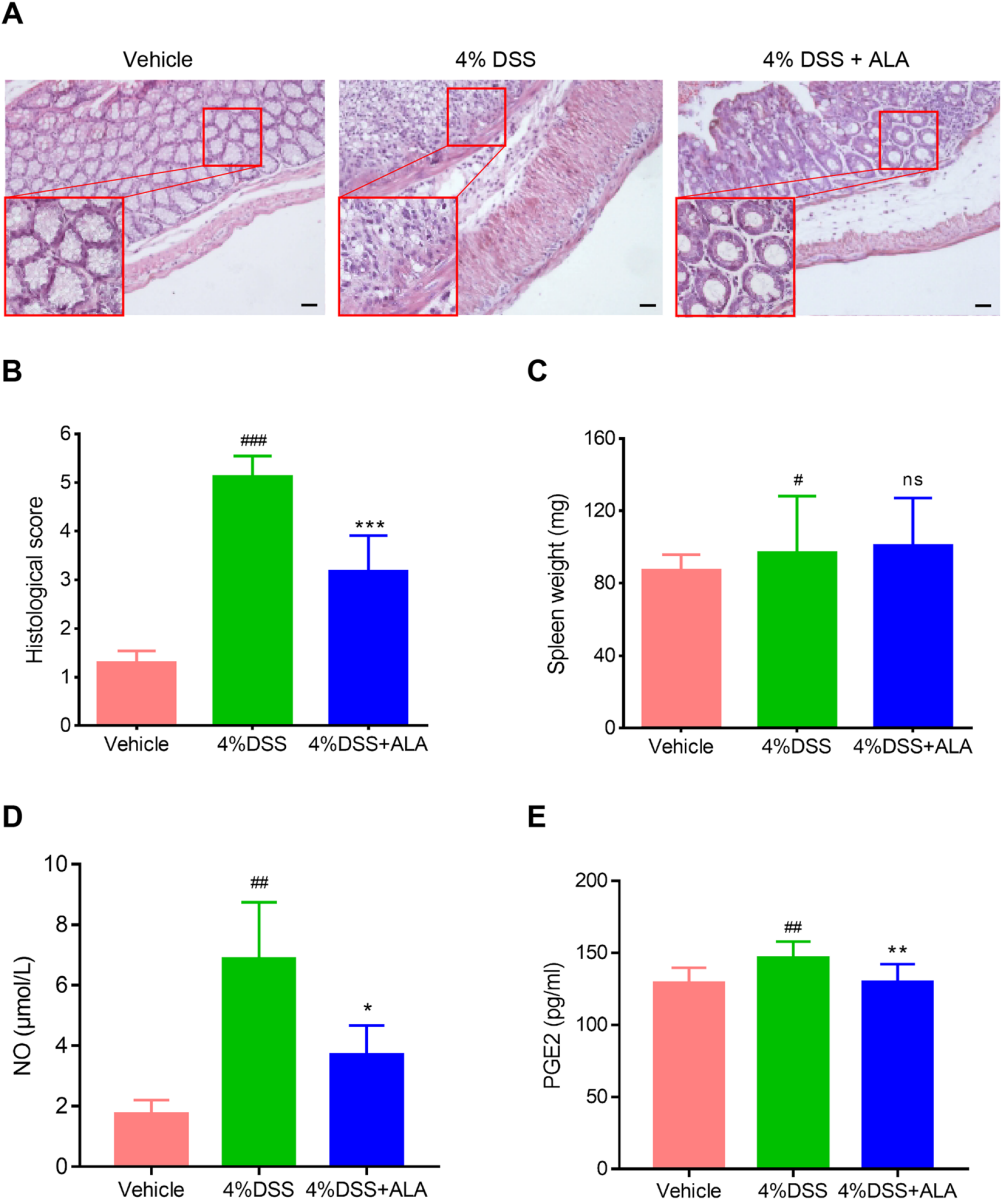
ALA ameliorated histopathological injury and inhibited the production of NO and PGE2 in DSS colitis mice. Representative H&E-stained colon sections (**A**) and histological score (**B**). Scale bar corresponds to 100 μm and applies throughout. (**C**) Spleen weight was measured at the end of mice study. The level of NO (**D**) and PEG2 (**E**) in serum was detected at the end of mice study. Data were expressed as the mean ± SD (n = 10 mice per group). *p < 0.05, **p < 0.01, ***p < 0.001 vs. DSS model group; ^#^P < 0.05, ^##^P < 0.01, ^###^P < 0.001 vs.control group; ns, no significance.

The DSS alone treatment group showed body weight loss, bloody diarrhea and colon shortening as compared with the control group, while ALA treatment obviously reversed DSS-induced body weight loss, bloody diarrhea and colon shortening (Fig. 1). Moreover, compared with the control group, the DSS model group exhibited severe neutrophil infiltration and histological injury; however, administration of ALA significantly attenuated the pathological changes induced by DSS (Fig. 2A,B). In our animal experiments, we failed to observe significant effects of ALA on DSS-colitis induced increase in spleen weight (Fig. 2C).

### ALA blocked NF-κB activation and downregulated NF-κB target genes in the colon

**Figure 3:**
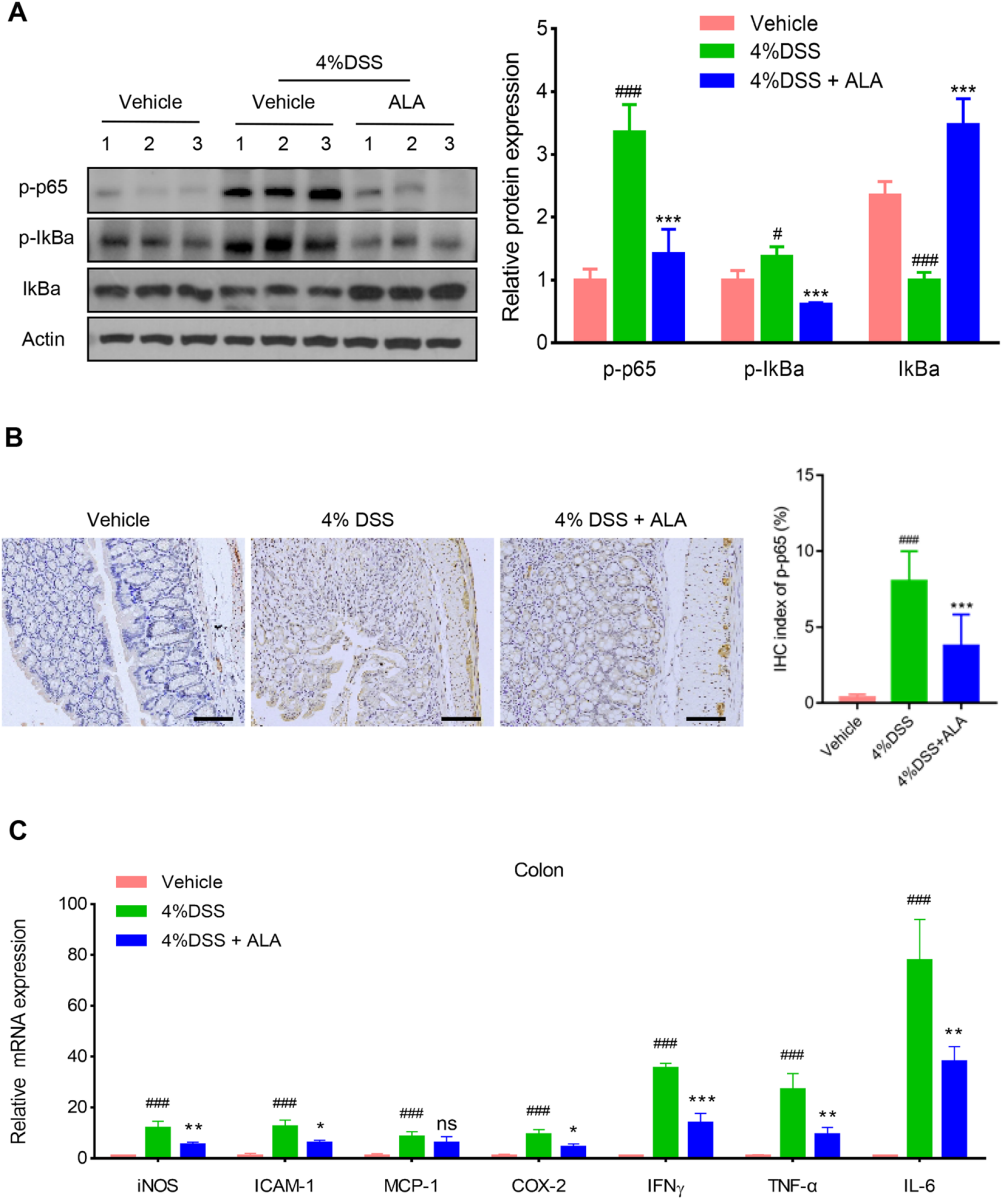
ALA inhibited NF-κB activation and target genes expression in DSS colitis mice. (**A**) Immunoblotting was performed with antibodies against mouse p-p65, p-IκBα, IκBα and β-actin. Quantification of the protein expression was performed by densitometric analysis of the blots. (**B**) Representative images of p-p65 immunostaining in colon tissue. Scale bar corresponds to 100 µm and applies throughout. (**C**) mRNA expression of the pro-inflammatory genes in colon tissue by qRT-PCR. Data were expressed as the mean ± SD of three independent experiments. n = 6 mice per group. *p < 0.05, ** p < 0.01, ***P < 0.001 vs. DSS model group; ^#^P < 0.05, ^###^P < 0.001 vs. control group; ns, no significance.

NF-κB is a major transcription factor of the pro-inflammatory signaling pathways participated in IBD^21^. We inferred that the anti-inflammatory effect of ALA on chemically induced IBD mice might be related to the blockade of NF-κB activation. Immunoblotting analysis indicated that DSS treatment led to a pronounced phosphorylation of NF-κB p65 and phosphorylation/degradation of IκBα in the colon of DSS model group, which was significantly suppressed by ALA treatment (Fig. 3A). On the other hand, phosphorylation of NF-κB p65 localization in the colon was detected by immunostaining. As expected, DSS treatment led to a pronounced increase of the NF-κB p65 positive signals in the colon tissue (Fig. 3B). However, NF-κB p65 positive signals in the colon were markedly reduced by ALA treatment. Furthermore, qPCR analyses of NF-κB target genes in the colon indicated a significant increase in the mRNA expression of iNOS, ICAM-1, MCP-1, COX-2, TNF-α, IFN-γ and IL-6 in the colon tissue of DSS model group compared with the control group (Fig. 3C). By contrast, administration of ALA reversed the increase in these inflammatory mediators induced by DSS treatment except for MCP-1. These findings indicate that the attenuated effect of ALA on DSS-induced colitis might be associated to the suppression of NF-κB signaling.

### ALA reduced the activity of MPO and the production of TNF-α, IL-6, NO and PEG2 in the colon

As a biomarker for leukocyte infiltration into inflamed tissue, MPO activity was determined. The results showed that the MPO activity in the colon tissue was markedly increased in mice receiving DSS alone treatment compared with control mice (Table 1). However, MPO activity in DSS model group was significantly reduced after ALA administration. Moreover, an obvious increase in the levels of TNF-α, IL-6, NO and PEG2 were detected in mice exposed to DSS compared to control mice (Table 1, Fig. 2D,E). Treatment with ALA resulted in a significant reduction in the levels of TNF-α, IL-6, NO and PEG2 in mice exposed to DSS. These results indicate that treatment with ALA reduces the activity of MPO and the production of pro-inflammatory mediators, and thereby suppresses inflammatory response.

**Table 1:** Effects of ALA on the activity of MPO and the levels of TNF-α and IL-6 in DSS colitis mice.

Group	MPO (U/mg pr.)	TNF-α (pg/mg pr.)	IL-6 (pg/mg pr.)
Vehicle	3.7 ± 0.4	15.6 ± 1.1	21.5 ± 2.9
DSS	16.4 ± 1.9^###^	140.8 ± 8.3^###^	224.6 ± 10.3^###^
DSS + ALA	10.9 ± 1.6**	66.2 ± 5.7***	86.2 ± 5.5***

The activity of MPO and the levels of cytokines were detected according to the specification of kits. Date were expressed as mean ± SD (n = 6 mice per group). ^###^p < 0.001 vs. control group; **p < 0.01, ***p < 0.001 vs. DSS model group.

### ALA restrained the activation of NF-κB in macrophages

**Figure 4:**
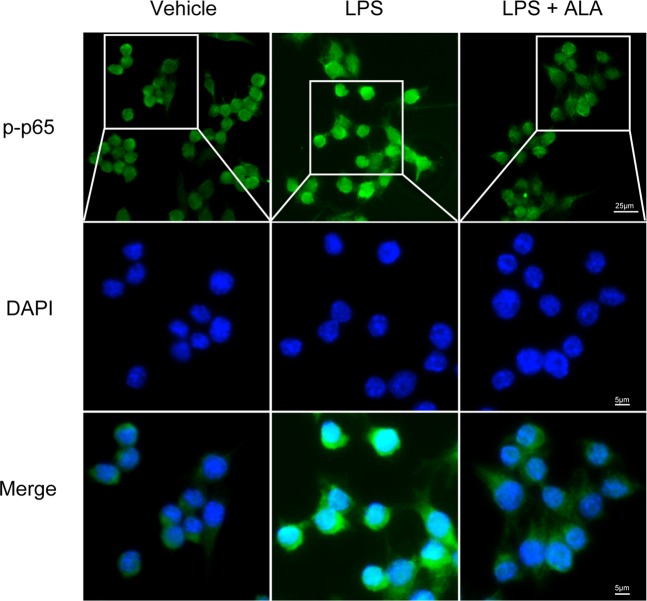
ALA inhibited NF-κB p65 activation *in vitro*. NF-κB p-p65 nuclear translocation in RAW264.7 cells was evaluated by immunofluorescence staining and images were captured by a fluorescence microscope as described in Methods. Scale bar corresponds to 25 μm or 5 μm as shown in our figure.

Immunofluorescence staining was used to further clarify the anti-inflammatory effect of ALA on NF-κB p65 activation in RAW264.7 cells. The results showed that LPS-induced p-p65 nuclear translocation was suppressed by ALA in RAW264.7 cells (Fig. 4).

### ALA inhibited NF-κB activity through activation of PXR

**Figure 5:**
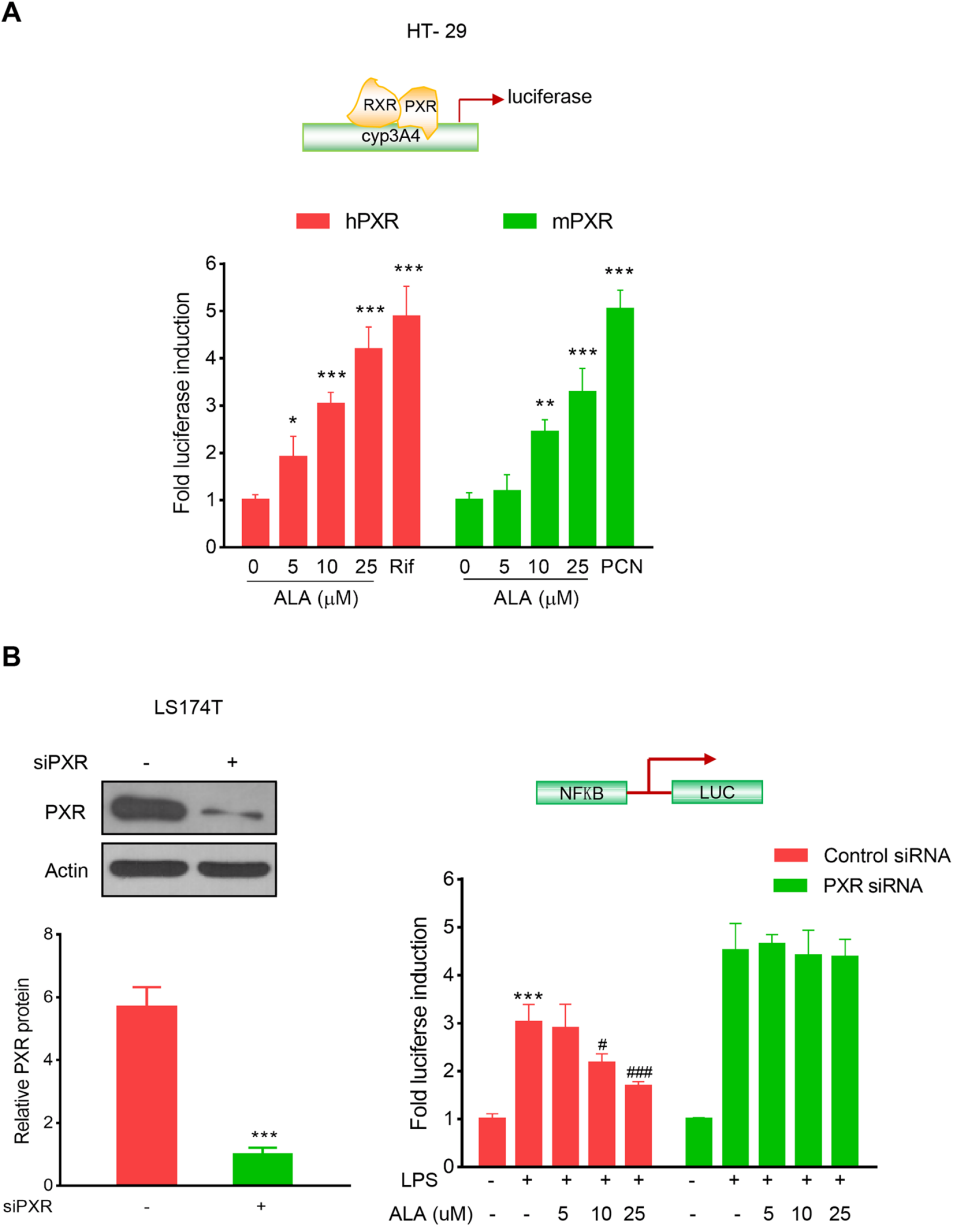
ALA activated h/m PXR and inhibited NF-κB-luciferase activity in a hPXR dependent manner. (**A**) HT-29 cells were co-transfected with CYP3A4-luciferase reporter combined with pRL-TK, and the wild-type hPXR expression construct (pSG5-hPXR) or the wild-type mPXR expression construct (pSG5-mPXR). Cells were incubated with ALA (0–25 μM) and rifampicin or ALA (0–25 μM) and PCN (5 μM) for 24 h. Cell extracts were assayed for luciferase activity. (**B**) LS174T cells were electroporated with the hPXR siRNA or the control siRNA. Cells were subjected to western blot analysis to determine protein expression (Left panel) or subjected to NF-κB reporter assay as described in Methods (Right panel). The results were presented as the mean ± SD of three independent experiments. *p < 0.05, **p < 0.01, ***p < 0.001 vs. control cells; ^#^P < 0.05, ^###^P < 0.001 vs. LPS alone treatment cells.

**Figure 6:**
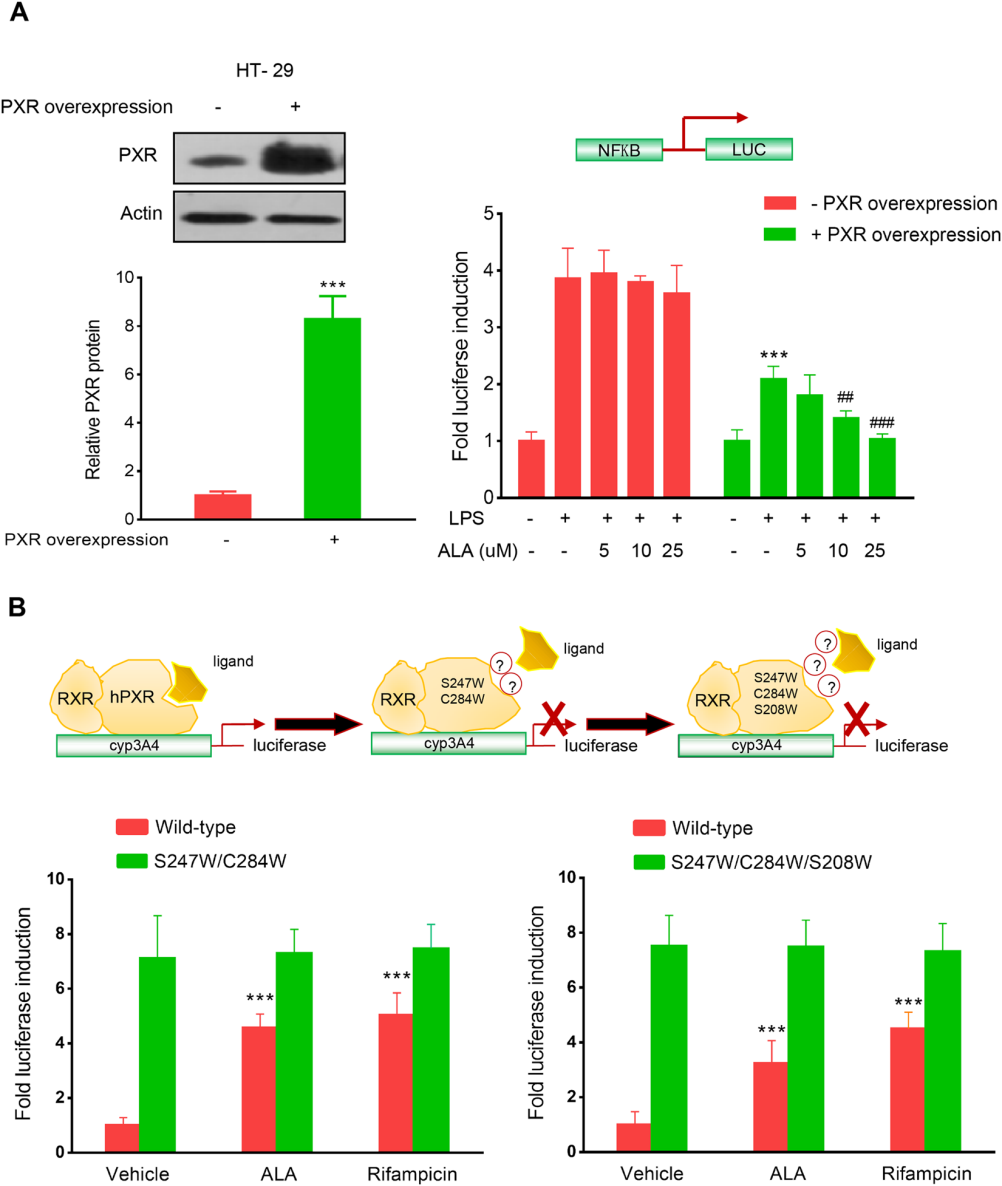
ALA enhanced hPXR-mediated NF-κB-luciferase activity inhibition, activated the wild-type hPXR but not the mutant hPXR. (**A**) HT-29 cells were electroporated with the hPXR expression vector pSG5-hPXR or the control vacuum vector. Cells were subjected to western blot analysis to determine protein expression (Left panel) or subjected to NF-κB reporter assay as described in Methods (Right panel). (**B**) HT-29 cells were co-transfected with CYP3A4-luciferase reporter combined with pRL-TK, and the wild-type hPXR expression construct (pSG5-hPXR) or the double-mutant (S247W/C284W) hPXR construct or the triple-mutant (S247W/C284W/S208W) hPXR construct. Cells were incubated with ALA (25 μM) or rifampicin (10 μM) for 24 h. Cell extracts were assayed for luciferase activity. The results were expressed as fold induction of the vehicle-treated cells. Data are presented as the mean ± SD of three independent experiments. ***p < 0.001 vs. control cells; ^##^P < 0.01, ^###^P < 0.001 vs. LPS alone treatment cells.

As shown in Fig. 5A, ALA dose-dependently induced both human and mouse (h/m) PXR transactivation in HT-29 colorectal cancer cells. Since our focus is on human cells, to explore whether PXR activation mediates the inhibition of NF-κB by ALA, we silenced hPXR gene in LS174T human colorectal cancer cells, a cell line with abundant hPXR expression. As expected, knockdown of hPXR in LS174T cells by hPXR siRNA significantly decreased hPXR protein (Fig. 5B, left panel). The hPXR-silenced LS174T cells were then transfected with a NF-κB-luciferase reporter and co-incubated with LPS and ALA. We observed that ALA dose-dependently decreased the LPS-induced NF-κB-luciferase activity in the control siRNA transfected LS174T cells; however, ALA did not decrease the LPS-induced activity of NF-κB-luciferase in the hPXR siRNA transfected LS174T cells (Fig. 5B, right panel). On the other hand, since HT-29 human colorectal cancer cells have less hPXR expression, we overexpressed hPXR in HT-29 cells (Fig. 6A, left panel). We observed that overexpression of hPXR in HT-29 cells inhibited the LPS-induced basal NF-κB-luciferase activity, and ALA treatment further enhanced the hPXR-mediated repression of NF-κB-luciferase activity (Fig. 6A, right panel). These results suggest that NF-κB activation suppression by ALA is hPXR dependent.

### Mutation of hPXR LBD disrupted the ALA-induced hPXR activation

To further evaluate whether ALA binds to hPXR LBD and subsequently activates hPXR, a transient transactivation reporter assay was performed by using the double-mutant construct (S247W/C284W) and the triple-mutant construct (S247W/C284W/S208W) in hPXR LBD, respectively. Previous studies have indicated that when hPXR LBD was effectively filled with either the double-mutant or the triple-mutant, it would not leave enough space for the established hPXR ligand SR12813 to bind to hPXR, which may ultimately lead to ligand-binding occlusion or ligand-independent constitutive activation^22,23^. Our results showed that both ALA and rifampicin activated the wild-type hPXR, whereas neither activated the double-or the triple-mutant (Fig. 6B). These findings indicate that ALA activates hPXR via binding to hPXR-LBD.

### ALA was docked to hPXR-LBD

**Figure 7:**
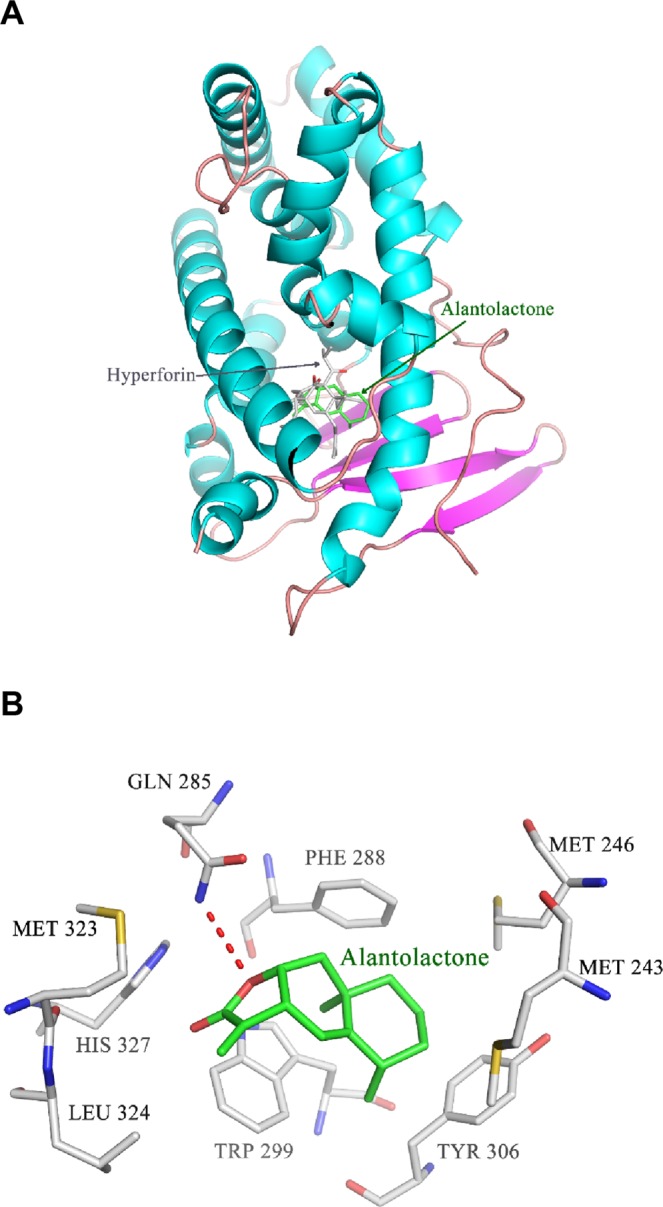
Molecular docking of ALA to the hPXR LBD. (**A**) ALA (shown in green stick) and the temple compound hyperforin (shown in grey stick) were docked into the hPXR LBD (shown in ribbon) using the MOE program as described in Methods. (**B**) ALA formed one hydrogen bond (shown in red dotted line) with GLN 285 and hydrophobic interactions with Met 243/246/323, Phe 288, Trp 299, Leu 324, and His 327. Atoms N, O and S were marked with blue, red and yellow, respectively.

The *in silico* modeling analysis indicated that the binding region of ALA was located below the hyperforin-binding site within hPXR receptor (Fig. 7A). The etheroxy-group of ALA formed one hydrogen bond with the nitrogen atom from the side chain amino-group of GLN 285 (Fig. 7B). In addition, ALA had hydrophobic interactions with residues Met 243/246/323, Phe 288, Trp 299, Leu 324, and His 327 in different directions, which may contribute to the high hPXR-binding affinity. These data confirmed the binding interaction between hPXR-LBD and ALA.

## Discussion

Since the exact etiology of IBD is still elusive, a number of animal models have been developed in order to study the possible mechanisms involved in the pathogenesis of IBD^24^. The most commonly used rodent model of IBD was developed by adding DSS in drinking water for about one week^2^. DSS has direct toxic effects on colonic epithelium and the acute colitis resembles human IBD^25^. In the present study, we found that ALA attenuated DSS-induced body weight loss, bloody diarrhea, colon shortening, inflammatory infiltration and histopathological injury in mice. However, ALA had no effects on DSS-induced splenomegaly, which was possibly due to the acute model of DSS colitis and further evaluation on this parameter using a chronic DSS colitis model is needed.

With increasing research conducted on IBD, possible mechanisms and important mediators involved in the pathogenesis of IBD have been disclosed. Among them, NF-κB activation has been linked to multiple aspects of the inflammatory process. As a major transcription factor, NF-κB regulates the expression of a diverse panel of genes, such as COX-2, iNOS, TNF-α, IL-1, adhesion molecules, and anti-apoptosis proteins^26^. Many of these genes are related to exacerbation of IBD. Thus, inhibition of NF-κB pathway represents a potential therapeutic strategy for IBD^21^. The established immunosuppressive drugs in IBD, such as corticosteroids, sulfasalazine, methotrexate and anti-TNF-a antibodies, are showed to mediate their anti-inflammatory effects at least partly via inhibition of NF-κB^27^. Although it was clearly showed that activated NF-κB contributes to intestinal inflammation, the biological functions of NF-κB are diverse in different cells and many of them have not been fully elucidated. In mucosal immune cells, elevated NF-κB activity is likely to trigger the expression of pro-inflammatory mediators and leads to more severe inflammation in IBD. On the other hand, the epithelial NF-κB appears to be a crucial adjustment factor for maintaining the integrity and homeostasis of intestinal mucosa barrier^28^. For instance, epithelial-specific inhibition of NF-κB through blocking IKKγ (NEMO) signaling pathway by IKKγ inhibitor, can spontaneously lead to severe enteritis^29^. Similarly, intestinal epithelial-specific NF-κB subunit RelA knock-out mice, shows the susceptibility to chemically induced acute colitis^30^. Hence, appropriate levels of NF-κB activity may be crucial for maintaining the intestinal immune homeostasis. In the current study, we found that ALA effectively decreased the activation of NF-κB in DSS colitis mice and RAW267.4 macrophage cells. In addition, ALA significantly reduced the expression of pro-inflammatory genes, the activity of MPO, and the production of TNF-α, IL-6, NO and PGE2 in DSS colitis mice. Thus, implying the anti-inflammatory effects of ALA on DSS colitis is associated with the suppression of NF-κB signaling pathway.

Recently, PXR was shown to inhibit NF-κB p65 from nuclear translocation, which is a basic step for NF-κB activation^31^. Additionally, activation of PXR suppresses the expression of NF-κB target genes including iNOS, IL-1α, IL-1β, IL-6 and TNF-α in the colon of DSS colitis mice^7^. Cheng *et al*. reported that treatment of PXR-humanized mice with rifaximin, a human-specific PXR agonist, results in significant inhibition of NF-κB and its target genes in the intestine in experimental mouse model of DSS colitis^32^. More recently, the same group reported that rifaximin compromises NF-κB signal and elicits protection in a PXR-humanized mouse model of colitis-associated cancer, which is associated with reduced inflammation, cancer cell proliferation, and pro-apoptosis^33^. In the current study, we found that ALA activated both mouse and human PXR; however, ALA could not activate the double-mutant (S247W/C284W) and the triple-mutant (S247W/C284W/S208W) of hPXR within the ligand-binding pocket. Molecular docking study confirmed the binding interaction between PXR-LBD and ALA. Furthermore, the hPXR knockdown and overexpression experiments demonstrated the effects of ALA on suppressing NF-κB activation was hPXR dependent.

In addition to its anti-inflammatory property, ALA has attracted interest as an anti-tumor agent in improving breast, lung, cervical and liver cancers in disease-associated cell models^15,34,35^. Notably, ALA was reported to induce oxidative DNA damage and prompt apoptosis in SW480 and SW1116 colorectal cancer cells^36^. It has been reported that patients with long-standing IBD are six times more likely to develop colorectal cancer than those normal people^37^. Further studies are required to elucidate the effects of ALA on abrogating colorectal cancer.

In summary, this is the first study to demonstrate that ALA may behave as a PXR ligand and decrease the susceptibility of mice to DSS-induced colitis via a mechanism associated with PXR-mediated suppression of NF-κB inflammatory signaling. The novel findings may contribute to the effective utilization of ALA or its derivatives in the management of human IBD.
